# Metabolic Syndrome and Its Components Predict the Risk of Type 2 Diabetes Mellitus in the Mainland Chinese: A 3-Year Cohort Study

**DOI:** 10.1155/2018/9376179

**Published:** 2018-12-13

**Authors:** Kun Wang, Qun-Fang Yang, Xing-Lin Chen, Yu-Wei Liu, Sheng-Shuai Shan, Hua-Bo Zheng, Xiao-Fang Zhao, Chang-Zhong Chen, Cheng-Yun Liu

**Affiliations:** ^1^Department of Geriatrics, Union Hospital, Tongji Medical College, Huazhong University of Science and Technology, Wuhan, China; ^2^Department of Hyperbaric Oxygen Center, Union Hospital, Tongji Medical College, Huazhong University of Science and Technology, Wuhan, China; ^3^Microarray Core Facility, Dana-Farber Cancer Institute, Harvard Medical School, Boston, MA 02215-5450, USA

## Abstract

**Introduction:**

It has well established that metabolic syndrome (MetS) can predict the risk of type 2 diabetes mellitus (T2DM) in some population groups. However, limited evidence is available regarding the predictive effect of MetS for incident T2DM in mainland Chinese population.

**Methods:**

A 3-year cohort study was performed for 9735 Chinese without diabetes at baseline. MetS and its components were assessed by multivariable analysis using Cox regression. Prediction models were developed. Discrimination was assessed with area under the receiver operating characteristic curves (AUCs), and performance was assessed by a calibration curve.

**Results:**

The 3-year cumulative incidence of T2DM was 11.29%. Baseline MetS was associated with an increased risk of T2DM after adjusting for age (HR = 2.68, 95% CI, 2.27–3.17 in males; HR = 2.59, 95% CI, 1.83–3.65 in females). Baseline MetS exhibited relatively high specificity (88% in males, 94% in females) and high negative predictive value (90% in males, 94% in females) but low sensitivity (36% in males, 23% in females) and low positive predictive value (31% in males and females) for predicting the 3-year risk of T2DM. AUCs, including age and components of MetS, for the prediction model were 0.779 (95% CI: 0.759–0.799) in males and 0.860 (95% CI: 0.836–0.883) in females. Calibration curves revealed good agreement between prediction and observation results in males; however, the model could overestimate the risk when the predicted probability is >40% in females.

**Conclusions:**

MetS predicts the risk of T2DM. The quantitative MetS-based prediction model for T2DM risk may improve preventive strategies for T2DM and present considerable public health benefits for the people in mainland China.

## 1. Introduction

Type 2 diabetes mellitus (T2DM) is one of the most common noncommunicable diseases worldwide. The International Diabetes Federation reported that 425 million adults suffer from diabetes worldwide, and this number will increase to 552 million by 2030 [1]. Rapid socioeconomic development and demographic changes, along with rapid westernization of diet and lifestyle [2], have led to the explosive increase in the prevalence of T2DM in China over the past few decades [3]. T2DM has become a major public health problem in China with a number of complications, such as heart disease, stroke, and diabetic nephropathy. Given the growing burden of this disease, early identification of individuals at high risk can help prevent, delay, and manage T2DM at an early stage. Prediction of T2DM can help guide interventions and health policy development.

Metabolic syndrome (MetS) is characterized by clustering factors, including overweight, raised blood pressure and blood glucose, and dyslipidemia. These factors are also potentially involved in the pathophysiology of T2DM [4–7]. Although the clinical application and practicality of MetS are still debatable [8], it has been proposed as a clinical tool to identify individuals predisposed to T2DM [9]. However, to date, only two studies in northern and eastern China have reported the predictive ability of MetS for T2DM based on relatively limited populations [10, 11].

In this study, we aimed to evaluate the performance of MetS and its components in predicting the risk of T2DM and to develop a quantitative MetS-based prediction model for the risk of T2DM in a mainland population from central China.

## 2. Methods

### 2.1. Study Population

The participants were community-dwelling residents who visited Wuhan Union Hospital for their annual health check-up between January 1, 2010 and July 30, 2010. Exclusion criteria were as follows: (1) subjects with baseline incomplete blood data, (2) individuals who was already with known diabetes mellitus, and (3) patients with previous clinical cardiovascular disease, stroke, or cancer. A total of 10,688 participants were enrolled in this cohort study.

### 2.2. Follow-Up Evaluations

**Figure 1:**
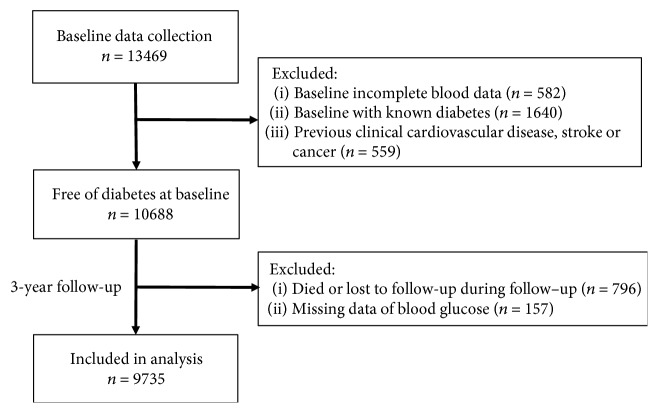
Flow chart of the 3-year cohort study.

We collected the health check-up data until October 31, 2013 by using the same baseline procedures. During follow-up, 953 individuals died, failed to follow-up, or showed missing pivotal data. A total of 9735 participants who completed at least 1 year of follow-up were included in the final analysis. Participants who developed T2DM during follow-up were considered incident T2DM cases, and the follow-up time for incident cases was calculated as the difference between the baseline and the examination when incident T2DM was initially identified. For participants who did not develop T2DM, the follow-up time was calculated as the difference between baseline and last known follow-up examination. A flow diagram illustrating patient selection is described in Figure 1.

The collection of health check-up data in Wuhan Union Hospital from January 1, 2010 to October 31, 2013 was approved by the ethics committee of Tongji Medical College, Huazhong University of Science and Technology, and complied with the Declaration of Helsinki of 2008. We verbally informed the participants that the data will be used anonymously for medical study. No informed consent was signed because the study was observational, and the data were anonymized.

### 2.3. Measurement of Variables

Trained investigators obtained demographic characteristics and previous medical histories through a standard questionnaire. Subjects underwent a brief physical examination that includes the measurement of height and weight. Height was measured to the nearest 0.5 cm and weight to the nearest 0.1 kg (Detecto Instrument, Webb City, MO). Body mass index (BMI) was calculated as weight in kilograms divided by the square of height in centimeters [12]. After a rest period of at least 5 min, the blood pressure of the subjects in the sitting position was measured by using a mercury sphygmomanometer according to a standardized protocol.

Blood samples were collected in the morning after an overnight fast and were processed within 2 h. After 75 g oral glucose tolerance test was administered, a second blood sample was drawn for glucose measurement. Automated chemistry analyzer (Beckman Coulter chemistry analyzer AU5800 series, Tokyo, Japan) was used for laboratory measurements, including the levels of plasma glucose, total cholesterol (TC), high-density lipoprotein cholesterol (HDLc), low-density lipoprotein cholesterol (LDLc), and triglycerides (TG).

### 2.4. Definition of Incident T2DM

Incident T2DM was defined as the presence of any of the following criteria at follow-up evaluation: (1) receiving oral hypoglycemic agents or insulin treatment, (2) fasting plasma glucose (FPG) ≥ 7.0 mmol/L (126 mg/dL), or (3) with plasma glucose ≥ 11.1 mmol/L (200 mg/dL) as evaluated by glucose tolerance test 2 hours after the oral dose.

### 2.5. Definitions of MetS

Asians are thought to have higher body fat percentage and cardiovascular risks than Caucasians at a given BMI [12]; therefore, the ATP III criteria for HDL cholesterol and waist circumference may not be appropriate for Asian populations. MetS was diagnosed in accordance with the diagnostic standard of Chinese Medical Association (CMA) Diabetes Branch. The participants were considered to exhibit MetS if they met three or more of the following criteria: (1) overweight: BMI ≥ 25.0 kg/m^2^; (2) impaired fasting glucose (IFG): FPG ≥ 6.1 mmol/L or impaired glucose tolerance (IGT): 2 hour postprandial plasma glucose (2hPG) ≥ 7.8 mmol/L or diagnosed diabetes; (3) hypertension: systolic/diastolic blood pressure ≥ 140/90 mmHg or taking antihypertensive agents; and (4) dyslipidemia: fasting triglycerides ≥ 1.7 mmol/L or fasting HDL-C < 0.9 and < 1.0 mmol/L in males and females, respectively.

### 2.6. Statistical Analysis

Summary statistics of the baseline characteristics of all patients and stratification by incidence of T2DM were expressed as means and standard deviations (SD) or medians and interquartile ranges for continuous variables and frequencies and proportions for categorical variables. Differences among groups were analyzed using one-way ANOVA, Kruskal-Wallis test, and chi-square test for normally distributed continuous, skewed continuous, and categorical variables, respectively (Table 1).

Multivariate Cox proportional hazard regression was used to estimate the age-adjusted hazard ratios (HRs) and 95% CI for the development of T2DM associated with MetS and its components (Table 2). Predictors of T2DM included MetS and its components. Sensitivities, specificities, and positive and negative predictive values (PPV and NPV) were also evaluated (Table 3).

**Figure 2:**
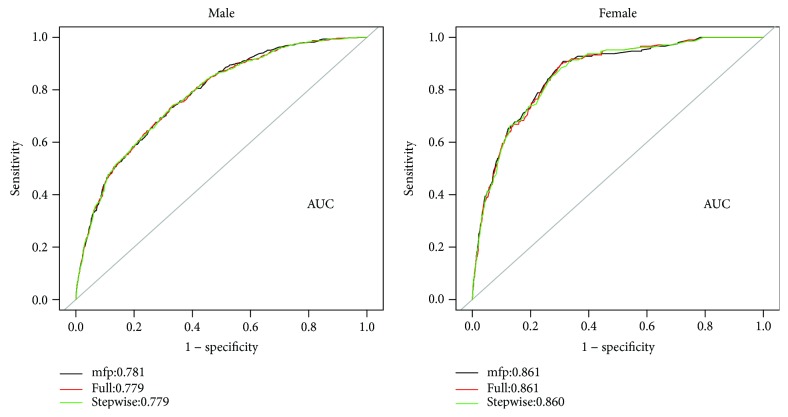
ROC curves of the components of MetS at baseline in predicting T2DM in man and woman (bootstrap resampling times = 500). AUC confidence interval and significance tests using bootstrap resampling. ROC: receiver operating characteristic curves; AUC: area under curve.

**Figure 3:**
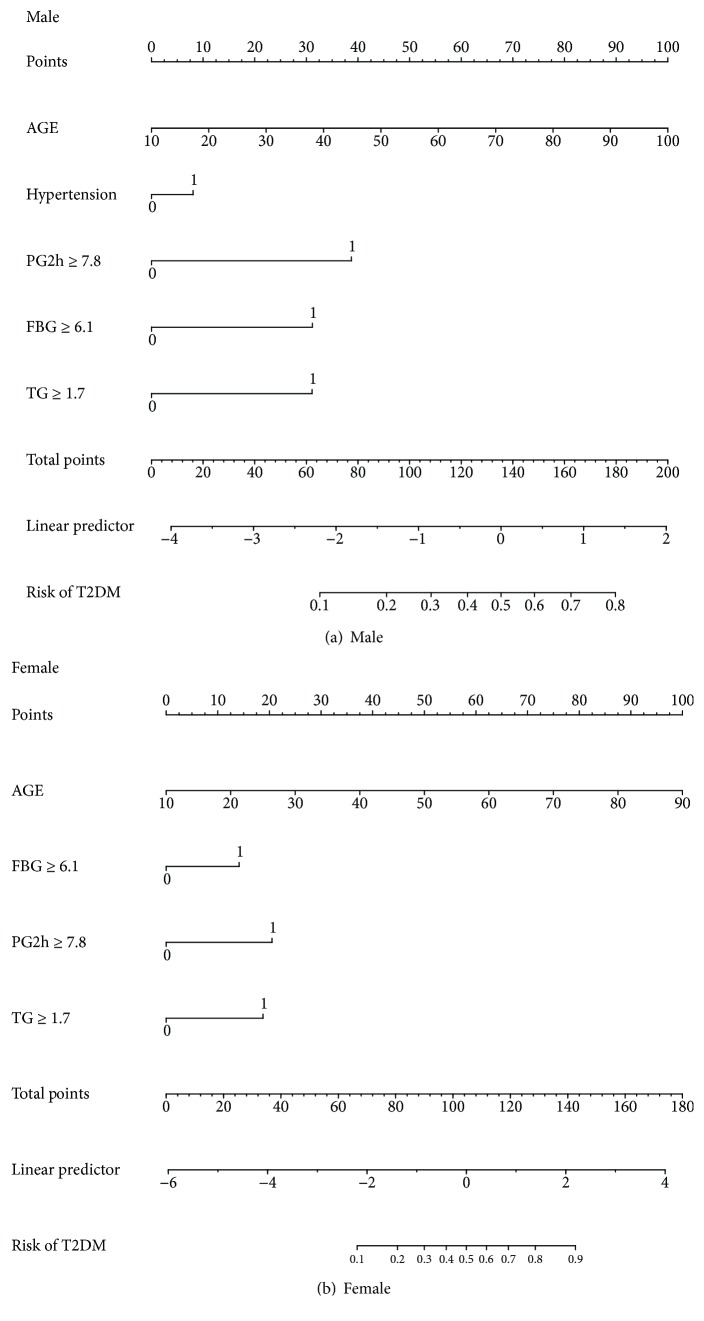
Nomogram to estimate the risk of T2DM using part of the components of MetS (stepwise model, bootstrap resampling times = 500). To use the nomogram, find the position of each variable on the corresponding axis, draw a line to the points axis for the number of points, add the points from all of the variables, and draw a line from the total point axis to determine the T2DM probabilities in 3 years at the lower line of the nomogram.

**Figure 4:**
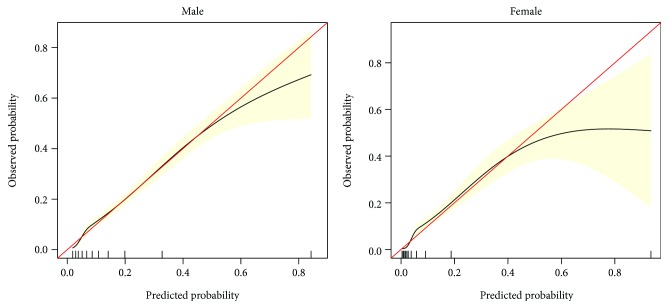
Calibration curves of the stepwise model nomogram (bootstrap resampling times = 500). On the calibration curve, *x*-axis is nomogram-predicted probability of incident T2DM in 3 years, and *y*-axis is observed incident T2DM in 3 years. The red line represents a perfect prediction by an ideal model. The black line represents the performance of the nomogram, which is a closer fit to the diagonal dotted line, representing improved prediction. The pink area is the 95% CI of the calibration curve.

Multivariable logistic regression analysis for the prediction model was performed. The following three kinds of prediction models were built: (1) full model, which includes age and all of the MetS components; (2) stepwise model, a backward step-down selection process that uses a threshold of *P* < 0.05 and excludes some factors without clinical significance (according to the Akaike information criterion); and (3) multivariable fractional polynomials (MFP) model [13], which performs nonlinear risk relationships between continuous variables and the outcome (Table 4). The receiver operating characteristic (ROC) curves of the three models were constructed using bootstrap resampling (times = 500) and are presented in Figure 2. By C statistics and DeLong algorithm [14], we found no significance between the three models in the capability to discriminate participants with and without incident T2DM (data not shown), so we chose the easiest “stepwise model” for the nomogram (Figure 3). The predictive accuracy of the nomogram was also measured by bootstrap (500 resample) method. Calibration curves [15, 16] were plotted to assess nomogram validation (Figure 4). The predicted probability of recurrence versus actual recurrence on the entire sample was compared using 500 bootstrap resamples to reduce over fit bias.

Score sheets to estimate absolute risk for the outcome were derived from the nomogram of the stepwise model (Table 5). The nomogram and calibration curves of full and MFP models are also presented in Supplementary Materials (Figures S1–S4).

All analyses were performed using statistical packages R (R Foundation; http://www.r-project.org; version 3.4.3) and EmpowerStats (http://www.empowerstats.com; X&Y Solutions Inc.). A value of *P* < 0.05 (two sided) was considered statistically significant.

## 3. Results

### 3.1. Baseline Characteristics and T2DM Incidence

Among the 9735 participants (aged 17–96 years) included in the final analysis, 60.48% were males, the mean (standard deviation) age of the cohort was 44.29 (13.52) years, and the baseline prevalence of MetS was 11.85% (1154). After an average observation period of 1.74 years, 11.29% (1099) of the participants developed T2DM.  Table 1 compares the baseline demographic and biochemical characteristics of individuals according to the presence or absence of T2DM incidence. Significant differences were observed among the groups.

T2DM incidence rates were 3.33% (321/9735), 4.01% (359/8949), and 6.65% (419/6300) in 2011, 2012, and 2013, respectively. This value also increased with age: 1.56% (22/1410) among the participants aged 17–29 years, 5.72% (138/2414) among those aged 30–39 years, 10.45% (303/2899) among those aged 40–49 years, 19.68% (356/1809) among those aged 50–59 years, 21.89% (141/644) among those aged 60–69 years, and 24.87% (139/559) among those aged >70 years.

### 3.2. Multivariate Analysis of Incident T2DM according to MetS and Its Components

Table 2 shows the results of the multivariate Cox proportional hazard models for T2DM prediction according to MetS and its individual components. After age adjustment, baseline MetS and its components (except Low HDLc) were all significantly positively associated with the risk of T2DM both in males and females. By contrast, low HDLc level (fasting HDL-C < 0.9 or< 1.0 mmol/L in males and females, respectively) at baseline exhibited no significant age adjusted association with T2DM incident (HR = 1.19, 95% CI, 0.67–2.11, *P* = 0.545 in males; HR = 1.74, 95% CI, 0.97–3.10, *P* = 0.0619 in females) but the associations still exhibited positively correlated trends.

### 3.3. Predictive Performance of MetS and Its Components for Risk of T2DM

Table 3 shows the predictive performance of MetS and its individual components at baseline in predicting the 3-year incidence of T2DM. We found that, both in males and females, almost all of the variables exhibited a relatively high specificity and a high negative predictive value but exhibited a low sensitivity and low positive predictive value. These results suggest that the absence of the MetS and its components at baseline may correctly identify the individuals free of incident T2DM.

### 3.4. The MetS-Based Prediction Model and Nomogram

Age and individual components of MetS were considered candidate variables for the prediction model. Three models that incorporated the above independent predictors were developed (bootstrap resampling times = 500). Full model includes age and all of the MetS components, stepwise model selects part of the candidate variables, and MFP model use fractional polynomials to model continuous risk variables, such as age. The formula and predictive performance of the three models both in males and in females are presented in Table 4. The AUCs for the three models in males and females were without exception >0.75, which indicated reasonable capabilities to discriminate participants with and without incident T2DM. The ROC curve (bootstrap resampling times = 500) of the three models both in males and in females are presented in Figure 2.

To provide a measurable tool for predicting individual probability of T2DM, we chose the most convenient “stepwise model” for the nomogram (Figure 3). The calibration curve of the nomogram for the probability of T2DM demonstrated good agreement between prediction and observation in males. As for woman, when the predicted probability > 40%, the predicted probability > observation probability, to be specific, when the predicted probability > 40% in woman, this nomogram is likely to overestimate the risk (Figure 4). The nomogram and calibration curves of full and MFP models are presented in Supplementary Materials (Figures S1–S4).

For increased convenience and facility, we further developed a point score system to estimate T2DM risk, this approach allows manual estimation of the 3-year risk of T2DM, as shown in Table 5. Ages younger than 50 and 55 years were considered referent categories in males and females, respectively. Other items are part of the components of MetS. Each item points to appropriate scores; after calculating the total item score, the risk of T2DM in males (A) and females (B) is shown in the unshaded area.

## 4. Discussion

In this population-based cohort study, MetS and its individual components (overweight, IFG, IGT, hypertension, TG ≥ 1.7 mmol/L, and low HDL) at baseline exhibited relatively high specificity and high negative predictive value for correctly identifying an individual with low T2DM risk. In addition, we developed a quantitative and easy-to-use prognostic nomogram integrating the MetS components and age at baseline to predict incident T2DM in 3 years. The nomogram showed relatively good predictive discrimination after internal validation.

Some studies have revealed the correlation between MetS and risk of T2DM. The Framingham study [17] in 2005 reported that MetS trait count was highly related to an increased risk in developing T2DM over 8 years of follow-up. The National Cholesterol Education Program of USA [9] reported that MetS can independently predict diabetes. Similarly, Ley et al. [18] and Hajat et al. [19] confirmed the value of MetS to identify individuals at risk of T2DM in aboriginal Canadians and Abu Dhabi, respectively. The prediction influence of MetS for diabetes was also identified among pediatric population [20] and the elderly [21]. Our findings are consistent with the previous studies. We further offered the prognostic nomogram and point score system prediction models to estimate T2DM risk based on the MetS components. This system is of particular interest for mainland Chinese communities with a high prevalence of T2DM.

In our study, we observed the baseline prevalence of MetS was 11.85%, and the 3-year cumulative incidence of T2DM was 11.29%. After age adjustment, MetS and its components (except low HDLc) at baseline were all significantly associated with an increased risk of T2DM in males and females. The high specificity and negative predictive values of MetS and its individual components at baseline in predicting T2DM incidence in our study are consistent with those in the study of Ley et al. [18] for correctly identifying disease-free individuals at follow-up. These findings suggest that individuals without MetS and its individual components possess low chance for developing future T2DM.

To provide a measurable tool that predicts the individual probability of T2DM, we calculated the prediction model by three methods and conducted a comprehensive assessment. Finally, we selected the stepwise model to develop the nomogram. We also found that this nomogram will overestimate the risk when the predicted probability is >40% in females. Calibration curves can help provide a relative real value. For instance, when a female is calculated at risk of 60% by this nomogram, her realistic risk should be approximately 50% according to the calibration curves. These efforts increase the credibility and practical value of our study.

Our study exhibits several strengths. First, the sample size was relatively large, and the health check-up participants were representative. Second, the quantitative prediction model is of greater clinical and social value than previous similar studies. Third, the comprehensive assessment of the three methods helps in selecting the most effective and easiest model.

Several limitations in this study are present. First, we were unable to collect interim data to analyze the time to onset of T2DM. Second, some participants missed follow-up, and some data were missing. Nevertheless, we still retained a high 3-year follow-up rate of 91.08% (9735/10688).

## 5. Conclusion

In summary, MetS is a simple method that can be used to predict the risk of T2DM. The quantitative MetS-based prediction model developed in this study can provide an individualized assessment for T2DM risk in the next 3 years. MetS can contribute to advanced intervention strategies to slow down T2DM progression and exhibits a certain degree of public health benefits in mainland China.

## Figures and Tables

**Table 1:** Baseline characteristics of participants included in the follow-up study according to the presence of incident T2DM.

Characteristic^∗^	Incident T2DM at 3-year follow-up		
	No (*n* = 8636)	Yes (*n* = 1099)	*P* value
Age (years)	43.21 ± 13.23	52.78 ± 12.72	<0.001
Gender, no. (%)			<0.001
Male	5087 (58.90%)	801 (72.88%)	
Female	3549 (41.10%)	298 (27.12%)	
BMI (kg/m^2^)	23.45 ± 3.16	25.33 ± 3.11	<0.001
Plasma glucose			
FBG (mmol/L)	4.92 ± 0.50	5.29 ± 0.68	<0.001
PG2h (mmol/L)	6.51 ± 1.25	7.68 ± 1.37	<0.001
Blood pressure			
Systolic (mm Hg)	116.33 ± 15.70	125.33 ± 16.97	<0.001
Diastolic (mm Hg)	77.52 ± 10.13	81.70 ± 10.56	<0.001
Pulse pressure (mm Hg)	38.81 ± 10.73	43.63 ± 12.66	<0.001
Lipid profile (mmol/L)			
Total cholesterol	4.77 ± 0.82	5.23 ± 0.92	<0.001
HDL cholesterol	1.54 ± 0.36	1.46 ± 0.36	<0.001
LDL cholesterol	2.47 ± 0.65	2.74 ± 0.71	<0.001
Triglycerides	1.22 (0.86–1.78)	1.91 (1.35–2.73)	<0.001
Metabolic syndrome, no. (%)	429 (5.6)	457 (29.3)	<0.001
Overweight	2231 (29.95%)	483 (50.47%)	<0.001
IGR	1551 (17.96%)	625 (56.87%)	<0.001
FBG ≥ 6.1 mmol/L	212 (2.48%)	163 (14.87%)	<0.001
PG2h ≥ 7.8 mmol/L	1452 (16.81%)	598 (54.41%)	<0.001
Hypertension	1876 (23.45%)	490 (47.90%)	<0.001
Dyslipidemia	2556 (29.98%)	591 (54.07%)	<0.001
TG ≥ 1.7 mmol/L	2361 (27.70%)	655 (59.93%)	<0.001
Low HDLc	297 (3.76%)	63 (6.46%)	<0.001

Data are shown as means ± SD, median (interquartile range), or no (%). T2DM: type 2 diabetes mellitus; IGR: impaired glucose regulation; FBG: fasting blood glucose; PG2h: 2-hour postprandial plasma glucose; TG: triglycerides; HDLc: high-density lipoprotein cholesterol. Overweight: defined as BMI greater than 25.0 kg/m^2^; hypertension: defined as systolic/diastolic blood pressure ≥ 140/90 mmHg or taking antihypertensive agents; low HDL cholesterol: defined as fasting HDL-C < 0.9 or <1.0 mmol/L in males and females, respectively. ^∗^The number of participants for each category varies slightly due to occasional missing values.

**Table 2:** Multivariate analysis of incident T2DM according to MetS and its components^∗^.

Variable	Male		Female	
	HR (95% CI) ^∗^	*P* value	HR (95% CI) ^∗^	*P* value
Metabolic syndrome	2.68 (2.27, 3.17)	<0.001	2.59 (1.83, 3.65)	<0.001
Overweight	1.76 (1.52, 2.04)	<0.001	1.94 (1.49, 2.52)	<0.001
IGR	3.87 (3.36, 4.46)	<0.001	3.61 (2.87, 4.55)	<0.001
FBG ≥ 6.1 mmol/L	3.12 (2.56, 3.79)	<0.001	3.05 (2.12, 4.38)	<0.001
PG2h ≥ 7.8 mmol/L	3.81 (3.32, 4.39)	<0.001	3.61 (2.87, 4.54)	<0.001
Hypertension	1.63 (1.40, 1.90)	<0.001	1.53 (1.15, 2.02)	0.0030
Dyslipidemia	2.38 (2.07, 2.75)	<0.001	2.18 (1.73, 2.75)	<0.001
TG ≥ 1.7 mmol/L	3.09 (2.66, 3.57)	<0.001	2.98 (2.36, 3.76)	<0.001
Low HDLc	1.19 (0.67, 2.11)	0.5452	1.74 (0.97, 3.10)	0.0619

Data are hazard ratio (95% CI), *P* value. T2DM: type 2 diabetes mellitus; MetS: metabolic syndrome; IGR: impaired glucose regulation; FBG: fasting blood glucose; PG2h: 2-hour postprandial plasma glucose; TG: triglycerides; HDLc: high-density lipoprotein cholesterol. Overweight: defined as BMI greater than 25.0 kg/m^2^; hypertension: defined as systolic/diastolic blood pressure ≥ 140/90 mmHg or taking antihypertensive agents; low HDL cholesterol: defined as fasting HDL-C < 0.9 or< 1.0 mmol/L in males and females, respectively. ^∗^Adjusted for age.

**Table 3:** Predictive value of baseline MetS and its components in predicting risk of T2DM at 3-year follow-up^∗^.

Variable	Male				Female			
	Sensitivity (%)	Specificity (%)	PPV (%)	NPV (%)	Sensitivity (%)	Specificity (%)	PPV (%)	NPV (%)
Metabolic syndrome	36	88	31	90	23	96	31	94
Overweight	54	61	18	89	39	83	16	94
IGR	59	80	31	92	52	85	23	95
FBG ≥ 6.1 mmol/L	16	97	48	88	11	98	33	93
PG2h ≥ 7.8 mmol/L	56	81	32	92	50	86	23	95
Hypertension	50	70	21	90	42	86	19	95
Dyslipidemia	58	63	20	90	43	80	15	94
TG ≥ 1.7 mmol/L	64	63	22	92	48	85	21	95
Low HDLc	2	99	15	87	4	97	11	92

MetS: metabolic syndrome; T2DM: type 2 diabetes mellitus; PPV: positive predictive value; NPV: negative predictive value; IGR: impaired glucose regulation; FBG: fasting blood glucose; PG2h: 2-hour postprandial plasma glucose; TG: triglycerides; HDLc: high-density lipoprotein cholesterol. Overweight: defined as BMI greater than 25.0 kg/m^2^; hypertension: defined as systolic/diastolic blood pressure ≥ 140/90 mmHg or taking antihypertensive agents; low HDL cholesterol: defined as fasting HDL-C < 0.9 or< 1.0 mmol/L in males and females, respectively. ^∗^Using bootstrap resampling (times = 500).

**Table 4:** Three prediction models for T2DM using components of MetS^∗^.

Model	AUC (95% CI)	Cut-off value	Sensitivity (%)	Specificity (%)	PPV (%)	NPV (%)
*Male*						
MFP	0.781 (0.762, 0.800)	−2.038	73	68	26	94
Full	0.779 (0.760, 0.799)	−2.071	74	67	26	94
Stepwise	0.779 (0.759, 0.799)	−2.064	74	67	26	94
*Female*						
MFP	0.861 (0.837, 0.885)	−2.925	91	69	18	99
Full	0.861 (0.838, 0.885)	−3.050	90	70	18	99
Stepwise	0.860 (0.836, 0.883)	−3.067	88	69	18	99

For males: MFP model: −1.92837-0.17487^∗^(age/100)^−2^ + 1.17798^∗^(PG2h ≥ 7.8 = 1) + 0.90866^∗^(T ≥ 1.7 = 1) + 0.97866^∗^(FBG ≥ 6.1 = 1) + 0.26414^∗^(hypertension = 1). Full model: −4.63421 + 0.03516^∗^age e + 0.23344^∗^(hypertension = 1) + 0.10789^∗^(overweight = 1) + 1.20251^∗^(PG2h ≥ 7.8 = 1) + 0.96130^∗^(FBG ≥ 6.1 = 1) + 0.95317^∗^(TG ≥ 1.7 = 1) + 0.00978^∗^(HDLc < 0.9 = 1). Stepwise model: −4.57730 + 0.03431^∗^age + 0.28763^∗^(hypertension = 1) + 1.20539^∗^(PG2h ≥ 7.8 = 1) + 0.96970^∗^(FBG ≥ 6.1 = 1) + 0.98210^∗^(TG ≥ 1.7 = 1). For females: MFP model: 0.98518–1.94843^∗^(age/100)^−1^ + 1.12166^∗^(PG2h ≥ 7.8 = 1) + 1.01383^∗^(TG ≥ 1.7 = 1) + 0.82966^∗^(FBG ≥ 6.1 = 1). Full model: −6.82459 + 0.07260^∗^age −0.07548^∗^(hypertension = 1) + 0.21320^∗^(overweight = 1) + 0.82601^∗^(FBG ≥ 6.1 = 1) + 1.14685^∗^(PG2h ≥ 7.8 = 1) −0.38866^∗^(HDLc <1.0 = 1) + 1.09116^∗^(TG ≥ 1.7 = 1). Stepwise model: −6.81323+ 0.07236^∗^age + 0.90655^∗^(FBG ≥ 6.1 = 1) + 1.17439^∗^(PG2h ≥ 7.8 = 1) + 1.12417^∗^(TG ≥ 1.7 = 1). AUC: area under curve. ^∗^Using bootstrap resampling (times = 500).

**Table:** (a) Prediction model for man

Item	Item score
(1) Age ≥ 50 years	44
(2) Hypertension	8
(3) 2-hour postprandial plasma glucose (2hPG) ≥ 7.8 mmol/L	37
(4) Fasting plasma glucose level ≥ 6.1 mmol/L	30
(5) Triglyceride ≥ 1.7 mmol/L	30

Total item score	3-year risk of T2DM
0~65	<10%
66~92	10%~20%
93~108	20%~30%
109~122	30%~40%
123~135	40%~50%
136~141	50%~60%
>141	>60%

**Table:** (b) Prediction model for woman

Item	Item score
(1) Age ≥ 55 years	57
(2) Fasting plasma glucose level ≥ 6.1 mmol/L.	14
(3) 2-hour postprandial plasma glucose (2hPG) ≥ 7.8 mmol/L	21
(4) Triglyceride ≥ 1.7 mmol/L	19

Total item score	3-year risk of T2DM
0~67	<10%
68~80	10%~20%
81~90	20%~30%
91~97	30%~40%
98~105	40%~50%
>105	>50%

## Data Availability

The data used to support the findings of this study are available from the corresponding author upon request.

## Supplementary Materials

Supplementary Materials Figure S1: the nomogram to estimate the risk of T2DM using part of the components of MetS (MFP model). Figure S2: calibration curves of the MFP model nomogram (bootstrap resampling times = 500). Figure S3: the nomogram to estimate the risk of T2DM using all of the components of MetS (full model). Figure S4: calibration curves of the full model nomogram (bootstrap resampling times = 500).
