# Supplementary material for: Metabolic Syndrome and Its Components Predict the Risk of Type 2 Diabetes Mellitus in the Mainland Chinese: A 3-Year Cohort Study
Source: Int J Endocrinol. 2018 Dec 13;2018:9376179. doi: 10.1155/2018/9376179 (PMC6311882; doi:10.1155/2018/9376179)
Supplement: Supplementary Materials — Figure S1: the nomogram to estimate the risk of T2DM using part of the components of MetS (MFP model). Figure S2: calibration curves of the MFP model nomogram (bootstrap resampling times = 500). Figure S3: the nomogram to estimate the risk of T2DM using all of the components of MetS (full model). Figure S4: calibration curves of the full model nomogram (bootstrap resampling times = 500). [file 9376179.f1.docx]

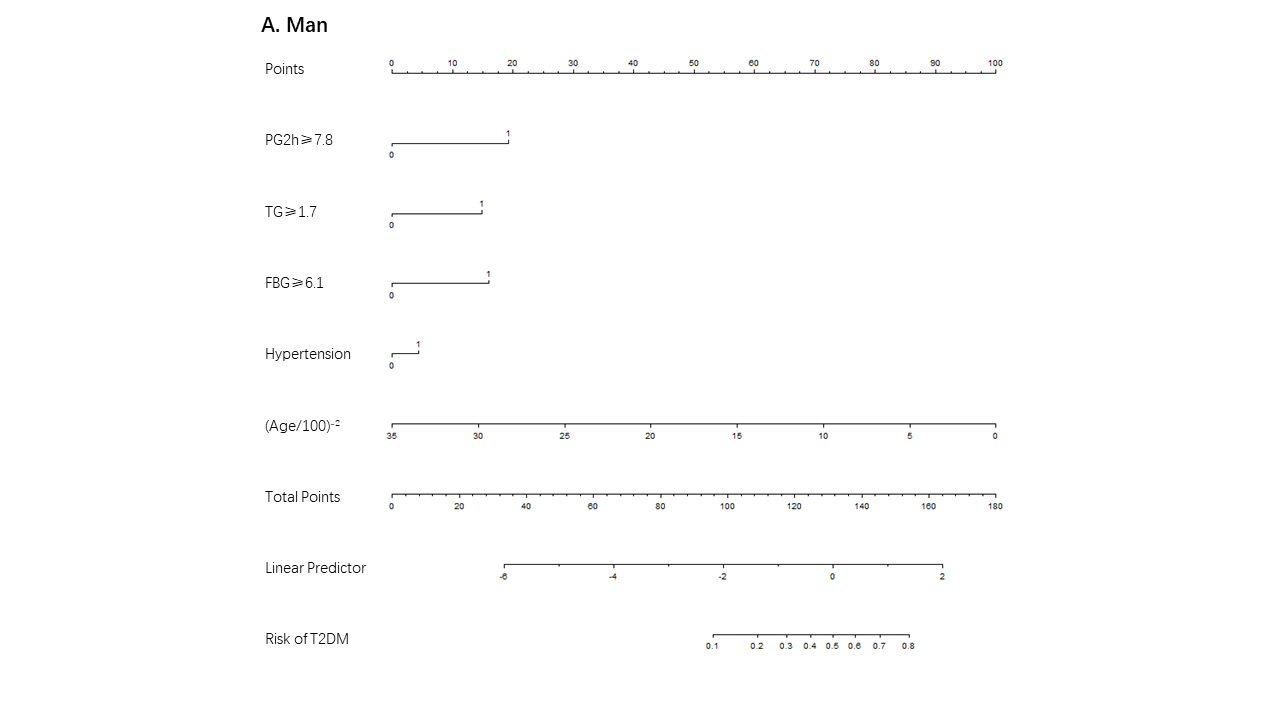

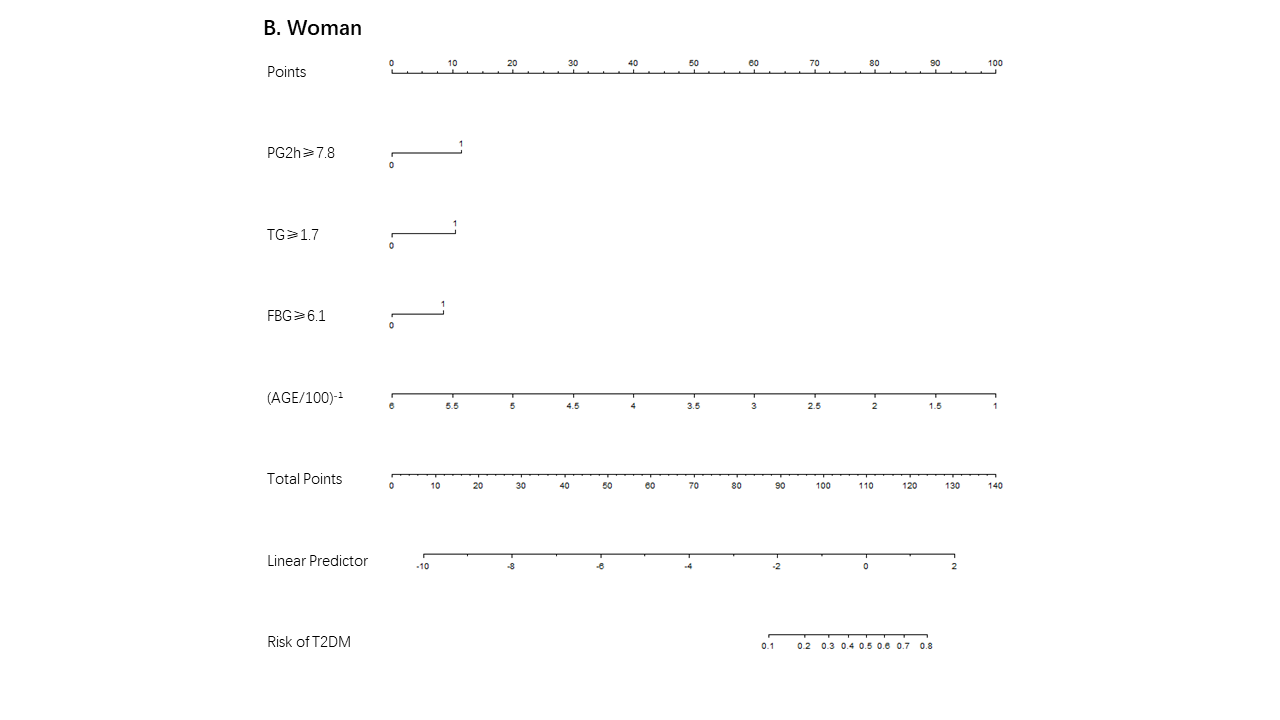


**Fig.S1 The nomogram to estimate the risk of T2DM using part of the components of MetS (MFP model)**. To use the nomogram, find the position of each variable on the corresponding axis, draw a line to the points axis for the number of points, add the points from all of the variables, and draw a line from the total points axis to determine the T2DM probabilities in 3 years at the lower line of the nomogram.


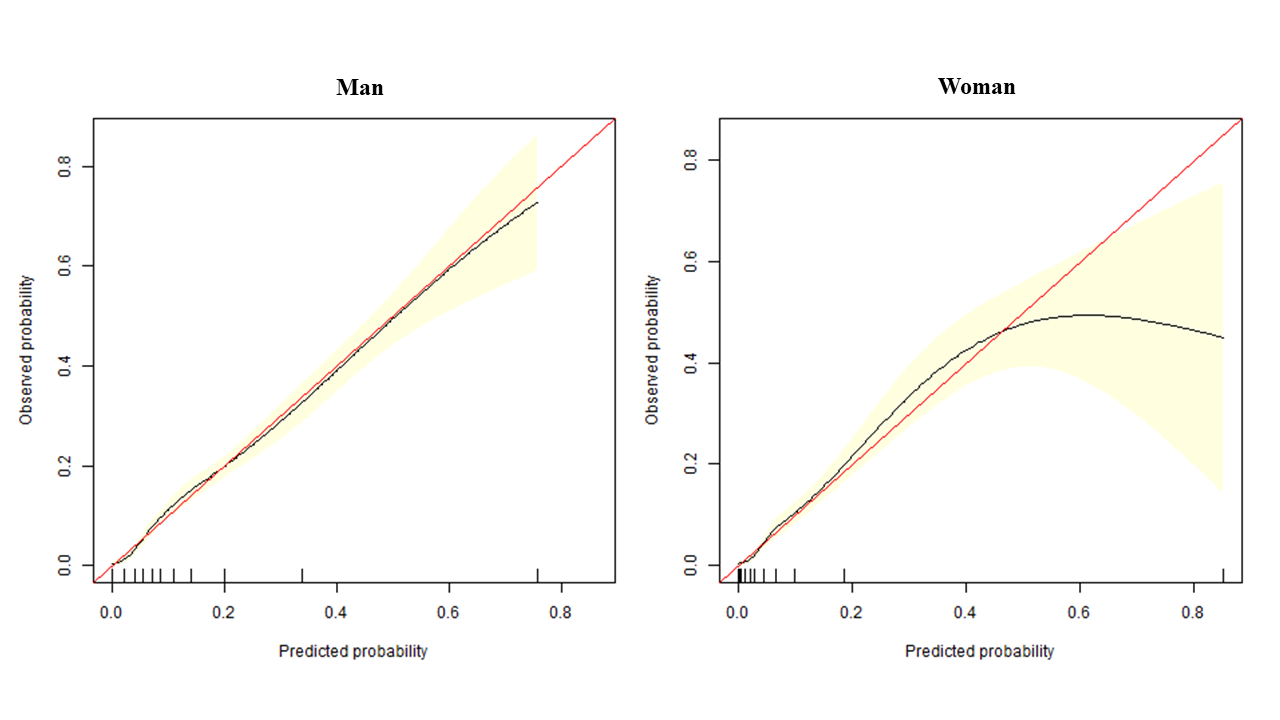


**Fig.S2 Calibration curves of the MFP model nomogram (Bootstrap resampling times = 500)** On the calibration curve, x-axis is nomogram-predicted probability of incident T2DM in 3 years, and y-axis is observed incident T2DM in 3 years.

The red line represents a perfect prediction by an ideal model. The black line represents the performance of the nomogram, of which a closer fit to the diagonal dotted line represents a better prediction. The pink area is the 95%CI of the calibration curve.


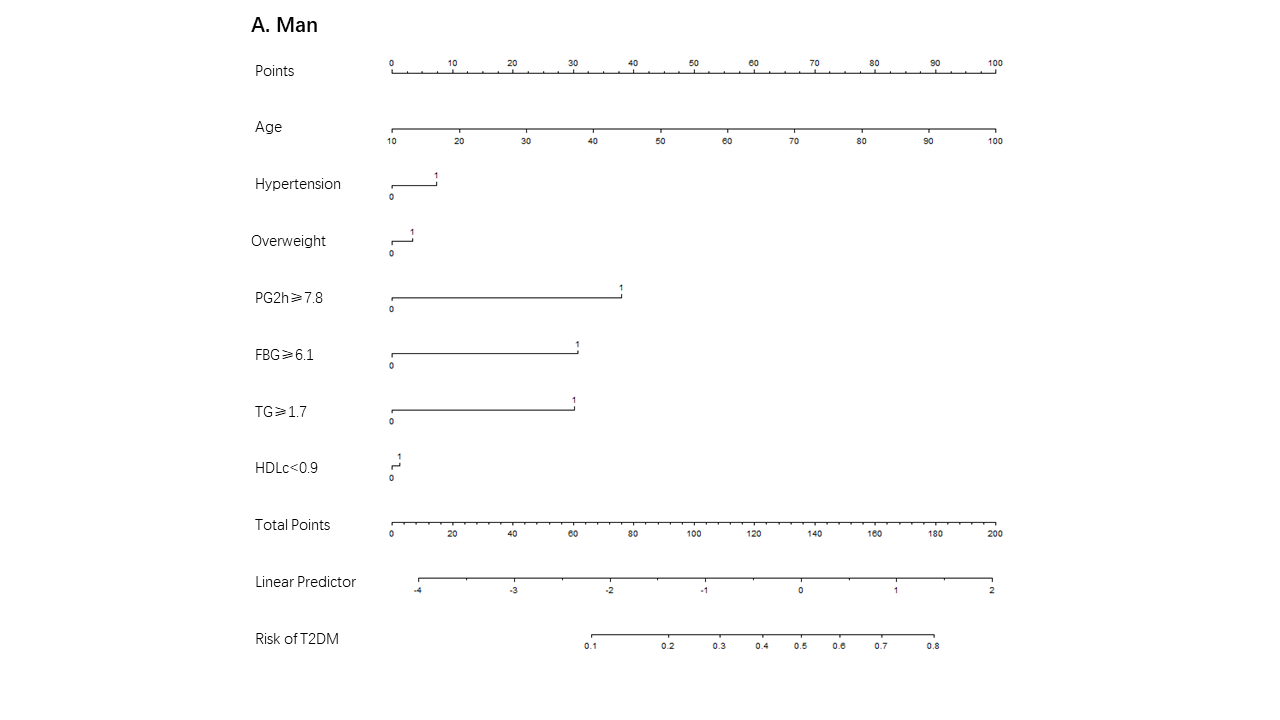

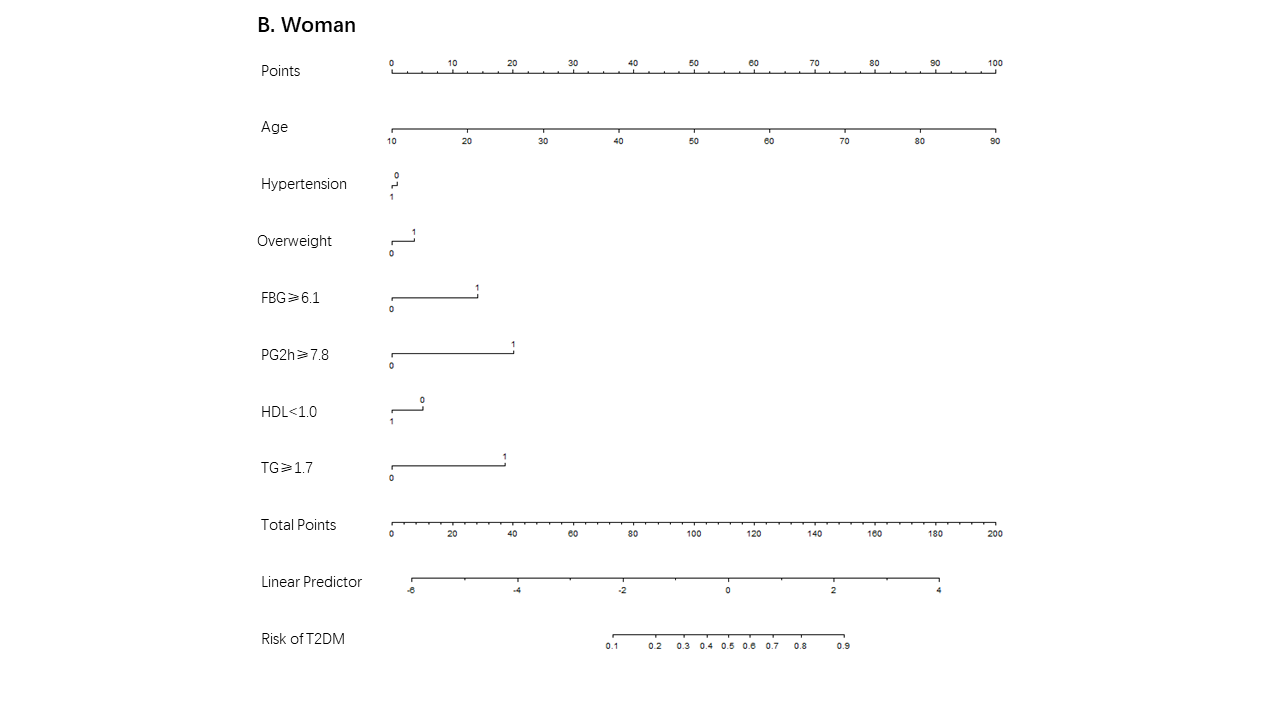


**Fig.S3 The nomogram to estimate the risk of T2DM using all of the components of MetS (Full model)**. To use the nomogram, find the position of each variable on the corresponding axis, draw a line to the points axis for the number of points, add the points from all of the variables, and draw a line from the total points axis to determine the T2DM probabilities in 3 years at the lower line of the nomogram.


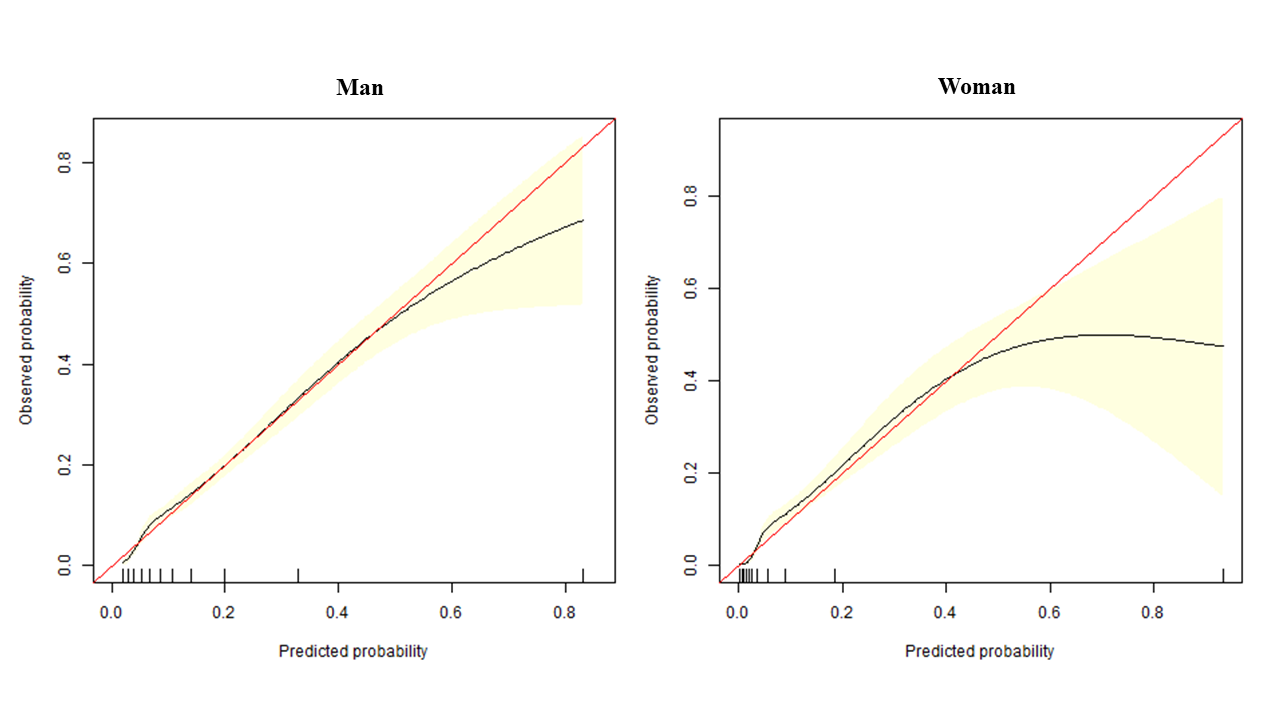


**Fig.S4 Calibration curves of the Full model nomogram (Bootstrap resampling times = 500)** On the calibration curve, x-axis is nomogram-predicted probability of incident T2DM in 3 years, and y-axis is observed incident T2DM in 3 years.

The red line represents a perfect prediction by an ideal model. The black line represents the performance of the nomogram, of which a closer fit to the diagonal dotted line represents a better prediction. The pink area is the 95%CI of the calibration curve.
